# Survival Factor A (SvfA) Contributes to *Aspergillus nidulans* Pathogenicity

**DOI:** 10.3390/jof9020143

**Published:** 2023-01-21

**Authors:** Joo-Yeon Lim, Ye-Eun Jung, Hye-Eun Hwang, Cheol-Hee Kim, Nese Basaran-Akgul, Sri Harshini Goli, Steven P. Templeton, Hee-Moon Park

**Affiliations:** 1 Department of Microbiology and Immunology, Indiana University School of Medicine-Terre Haute, Terre Haute, IN 47807, USA; 2 Laboratory of Cellular Differentiation, Department of Microbiology and Molecular Biology, College of Bioscience and Biotechnology, Chungnam National University, Daejeon 34134, Republic of Korea; 3 Laboratory of Developmental Genetics Department of Biology, College of Bioscience and Biotechnology, Chungnam National University, Daejeon 34134, Republic of Korea

**Keywords:** *Aspergillus nidulans*, survival factor A, pathogen-associated molecular pattern (PAMP), chronic granulomatous disease mice, lung immune response, inflammatory cytokines

## Abstract

Survival factor A (SvfA) in *Aspergillus nidulans* plays multiple roles in growth and developmental processes. It is a candidate for a novel VeA-dependent protein involved in sexual development. VeA is a key developmental regulator in *Aspergillus* species that can interact with other velvet-family proteins and enter into the nucleus to function as a transcription factor. In yeast and fungi, SvfA-homologous proteins are required for survival under oxidative and cold-stress conditions. To assess the role of SvfA in virulence in *A. nidulans*, cell wall components, biofilm formation, and protease activity were evaluated in a *svfA*-gene-deletion or an *AfsvfA-*overexpressing strain. The *svfA*-deletion strain showed decreased production of β-1,3-glucan in conidia, a cell wall pathogen-associated molecular pattern, with a decrease in gene expression for chitin synthases and β-1,3-glucan synthase. The ability to form biofilms and produce proteases was reduced in the *svfA*-deletion strain. We hypothesized that the *svfA*-deletion strain was less virulent than the wild-type strain; therefore, we performed in vitro phagocytosis assays using alveolar macrophages and analyzed in vivo survival using two vertebrate animal models. While phagocytosis was reduced in mouse alveolar macrophages challenged with conidia from the *svfA*-deletion strain, the killing rate showed a significant increase with increased extracellular signal-regulated kinase ERK activation. The *svfA*-deletion conidia infection reduced host mortality in both T-cell-deficient zebrafish and chronic granulomatous disease mouse models. Taken together, these results indicate that SvfA plays a significant role in the pathogenicity of *A. nidulans*.

## 1. Introduction

*Aspergillus nidulans* has been a model organism for studying the development, cell cycle, and cytoskeleton functions of filamentous fungi for over half a century [1,2,3]. *Aspergillus* species, which are saprotrophic fungi, are found in nature and grow on decaying vegetation. Typically, they do not cause any harm. In immunocompetent individuals, conidia are effectively removed by mucosal clearance and respiratory innate immune cells, such as neutrophils, alveolar macrophages, and dendritic cells [4,5]. In immunocompromised patients, however, *Aspergillus* species can cause fungal diseases, such as invasive aspergillosis (IA). *Aspergillus fumigatus* is the most common species causing IA (90% of human *Aspergillus* infections) [6], whereas much less attention has been given to *Aspergillus nidulans* as an opportunistic pathogen.

Patients with chronic granulomatous disease (CGD), who have defects in effective clearance and suffer from life-threatening bacterial and fungal infections, are at greater risk of *A. nidulans* infection than other immune-compromised patients [7]. CGD is a rare genetic disorder in which phagocytes fail to produce superoxide due to defects in one of the components of the NADPH oxidase complex: the two membrane-bound subunits gp91phox and p22phox and the three cytoplasmic subunits p47phox, p67phox, and p40phox [8]. The generation of oxidative products is one of the host defense mechanisms required for phagocytes to kill invading microorganisms and is also required for the assembly and formation of a functional NADPH oxidase on the phagosomal membrane [9,10].

While *A. fumigatus* and *A. nidulans* are responsible for 44% and 23% of CGD patients, respectively, approximately 30% of CGD patients show mortality with *A. nidulans* infection [7]. Although *A. nidulans* is more virulent than *A. fumigatus* based on mortality rates and propensity to spread [11,12], studies on IA caused by *A. nidulans* are lacking.

The fungal cell wall, which provides protection against external stresses and mediates interactions with external stimuli, is an essential element of the fungus. The fungal cell wall can be divided into two main structures: the outer and inner cell wall layers. The major components of the inner cell wall layer are chitin and β-glucan, which are well-conserved among fungal species [13]. The outer cell wall layer is variable across fungal species and contains melanin, hydrophobins, and proteins [13]. Cell wall components are considered pathogen-associated molecular patterns (PAMPs) [14]. The recognition of fungal-specific features by pattern recognition receptors (PRRs) on host immune cells is important for mounting a defense response [15]. Following the recognition of invading microorganisms, macrophage function can be activated depending partially on the phosphorylation of mitogen-activated protein kinases (MAPKs), including extracellular signal-regulated kinases (ERKs) and p38 [16]. Activated MAPKs translocate to the nucleus to induce the expression of pro-inflammatory genes [17].

Fungi grow in colonies via multicellular hyphae embedded in an extracellular matrix (ECM) [18]. This hyphal growth is consistent with the definition of a biofilm [19]. Typically, biofilms are a structural community of microbial cells. The chronic form of aspergillosis results in the formation of aspergilloma or fungal balls. These structures are composed of hyphae and conidiophores that grow as biofilms in the extracellular matrix [20,21]. In *Aspergillus* infection, hyphae form biofilms, including galactomannan (GM), galactosaminogalactan (GAG), α-1,3-glucans, and proteins, which exhibit cohesion properties and promote infection [18]. Biofilms shield fungi from attack by host immune cells and resist antifungal therapies [22].

Fungi produce a range of proteases that enable nutrient acquisition in the environment, including in host tissues, during infection [23]. Protease activity is commonly observed in various environmental allergens that induce asthma [24]. Asthma is a chronic lung inflammatory disorder caused by an immune response to inhaled allergens, causing an increase in mucous production, leukocyte infiltration, and collagen deposition [25].

In a previous study, we identified SvfA as a global transcription factor VeA-dependent protein that has only one domain: a yeast survival factor1 (Svf1) homologous protein. SvfA plays a role in the growth and development of *A. nidulans*; it regulates vegetative growth, functions in oxidative and cold stress responses, and affects conidial germination, conidial production, and completion of sexual development [26]. *A. nidulans* can produce two forms of the SvfA protein; only the production of the larger form is sexual-development-specific [26]. In *Saccharomyces cerevisiae*, the survival factor, functionally complemented by expression of mammalian B-cell lymphoma-extra large (Bcl-x_L_), inhibits reactive oxygen species generation and promotes survival under stress conditions [27,28]. Bcl-x_L_ regulates the intrinsic pathway of apoptotic cell death under prolonged oxidative and endoplasmic reticulum stress [29]. Yeast survival factor is also known to regulate cell survival by affecting sphingolipid metabolism [30]. The Svf1 homologous proteins are also found in plant-pathogenic fungi, such as *Fusarium graminearum* [31] and *Sclerotiana sclerotiorum* [32], and involved in both oxidative-stress response and pathogenicity.

To investigate the cellular function of SvfA on fungal virulence in *A. nidulans*, we investigated the immune response of alveolar macrophages (AMs) to conidia, biofilm formation ability, protease activity, and relative mRNA transcript levels involved in these cellular mechanisms using previously constructed *svfA*-deletion and -overexpression strains [26]. In addition, we reported the virulence of genetically engineered SvfA strains in experimental infection with immunodeficient zebrafish (*foxn1* morphant) and CGD mice (gp91^phox-/-^ mice). Foxn1, a forkhead box protein N1, is a transcription factor required for thymic epithelial cell development [33], and Foxn1 morphant can be used as a T-cell-deficient zebrafish model. SvfA deletion resulted in increased susceptibility to killing by AMs, decreased cell wall components, and decreased biofilm formation ability, suggesting that SvfA deletion attenuates virulence. Here, we present, for the first time, a novel role of SvfA in the pathogenicity of *A. nidulans*.

## 2. Materials and Methods

### 2.1. Generation of Fungal Strains

*A. nidulans* wild-type (WT), *svfA*-deletion (Δ*svfA*), and complementary (C’*svfA*) strains were obtained from our previous studies and maintained in *Aspergillus* glucose minimal medium (GMM) [26,34]. The C’*svfA* strain was generated by the re-introduction of the *svfA* gene to the Δ*svfA* strain [26]. To generate an Af*svfA*-overexpressing (OEAf*svfA*) strain [*yA2*; *argB2*; *pyroA4*; Δ*svfA*::*argB*; *pyroA*::*niiA*(p)::*AfsvfA*::*trpC*(t)], we performed BLASTP analysis of the *A. fumigatus* genome database using *A. nidulans* SvfA as a query and identified AfSvfA (Afu5g11820) as the best hit. The Af*svfA* open reading frame was cloned into pHS11 containing the *niiA* promoter. The resulting plasmid was then introduced into the Δ*svfA* strain. The induction of the Af*svfA* gene was analyzed with reverse transcriptase quantitative polymerase chain reaction (RT-qPCR) and phenotypic analysis (Supplementary Figure S1). After 48 or 72 h cultivation of the strains at 37 °C, spores were harvested using 0.08% Tween 80.

### 2.2. Biofilm Formation Assay

Fungal biofilm assays were performed using 12-well plates, as described previously [34]. A total of 10^5^ conidia in 1 mL of GMM were incubated in a 12-well plate for 16 h at 37 °C and washed thrice with PBS. The plates were stained with 1 mL of 0.01% (w/v) crystal violet solution for 6 h. Destaining with 1 mL of 30% acetic acid was conducted. Adhesion capacity was quantified by measuring the absorbance at 550 nm using a spectrophotometer.

### 2.3. Protease Activity

For the halo assay, 5 × 10^3^ conidia were point-inoculated on Czapek–Dox medium containing 1% skim milk powder instead of sodium nitrate and incubated for 3 d at 30 °C. An azocasein assay was performed as previously described, with some modifications [34,35]. Cells were grown in Czapek–Dox broth containing 1% skim milk powder for 3 d at 37 °C, and the supernatants were used as a crude enzyme source. Four volumes of ice-cold acetone were added and kept in a 4 °C cold room overnight. Protein pellets were collected by centrifugation for 20 min at 4 °C and air-dried. Protein concentrations were calculated using the BCA Protein Assay Kit (Thermo Fisher Scientific, Waltham, MA, USA) according to the manufacturer’s protocol. Azocasein (Sigma, St. Louis, MO, USA) was dissolved at a concentration of 5 mg/mL in an assay buffer containing 50 mM Tris (pH 8.0), 0.2 M NaCl, 5 mM CaCl_2_, and 0.05% Triton X-100. Supernatants from the cultures (200 µL) were mixed with 500 µL azocasein solution and incubated for 90 min at 30 °C. The reactions were stopped with the addition of 200 µL of 12% (*v*/*v*) trichloroacetic acid. The reaction mixtures were left at 25 °C for 30 min and centrifuged at 8000× *g*. The supernatant (200 µL) of the reaction mixture was mixed with 200 µL of 1 M NaOH. The absorbance of the released azo dye was measured at 436 nm.

### 2.4. RNA Preparation, cDNA Synthesis, and Quantitative Real-Time PCR

Cells at each developmental stage were ground using liquid nitrogen with a pestle and mortar [36]. Total RNA was extracted using TRIzol reagent according to the manufacturer’s protocols (Invitrogen, Waltham, MA, USA). cDNA was synthesized using 4 μg of extracted RNA, hexamer primers, and M-MLV reverse transcriptase (Enzynomics, Daejeon, Republic of Korea), as described in the manufacturer’s instructions. RT-qPCR was performed using a Bio-Rad CFX96 Real-Time PCR System (Bio-Rad, Hercules, CA, USA) and TOPrealTM qPCR 2X PreMIX Kit (Enzynomics, Daejeon, Republic of Korea). Transcript levels of target genes were normalized against those of 18S rRNA using the 2^−ΔCt^ method, as described previously [37]. The primers used for RT-qPCR are listed in Supplementary Table S1.

### 2.5. Phagocytosis and Macrophage Killing Assay

The mouse alveolar macrophage MH-S cell line (ATCC CRL-2019) was cultured in RPMI-1640 medium supplemented with 0.05 mM 2-mercaptoethanol and 10% fetal bovine serum (FBS) and incubated at 37 °C in 5% CO_2_ [38].

MH-S cells (10^6^ macrophages/well) were plated on 12-well cell culture plates with cover glasses and incubated for 2 h at 37 °C in 5% CO_2_. The cells were stimulated with 3-fold conidia for 2 h and washed thrice with Dulbecco’s phosphate-buffered saline (DPBS). Macrophages and conidia were co-cultured for an additional 3 h. The wells were then washed with DPBS and stained with calcofluor white (CFW, 1 μg/mL in DPBS) to label extracellular conidia [34]. The number of conidia phagocytosed by the macrophages was quantified as the percentage of macrophages containing at least one ingested conidium. The phagocytic index was calculated as the average number of ingested conidia per phagocytosing macrophage.

Assays for macrophage killing of conidia were performed as previously described with some modifications [39,40]. Briefly, MH-S cells (10^6^ macrophages/well) were stimulated with 10-fold conidia for 2 h and washed thrice with DPBS to remove unbound conidia. The cells were subsequently incubated for 0 and 16 h at 37 °C in 5% CO_2_. After incubation, the macrophages were lysed with 1 mL of distilled water, and conidia were harvested. The conidial supernatant was serially diluted in the RPMI medium and plated on GMM agar supplemented with 0.005% Triton X-100. Colony-forming units (CFUs) were counted after incubation for 3 d at 37 °C. The percentage of dead conidia (the difference between the number of CFUs in the lysate at 0 and 16 h per the number of CFUs in the lysate at 0 h) was calculated.

### 2.6. Microscopy

For microscopic observations, an Olympus System microscope Model BX51 (Olympus) equipped with UPlanSApo 60× and UPlanFL 100× objective lenses (Olympus, Shinkjuku, Japan) and a stereomicroscope Model SMZ800 (Nikon, Minato, Japan) were used. Images were captured with a DP71 digital camera (Olympus) and processed using DP manager imaging software (Olympus, Shinkjuku, Japan).

### 2.7. Polysaccharide Analysis

The amount of β-1,3-glucan in conidia was measured using an enzymatic yeast beta-glucan kit, following the manufacturer’s protocol (Megazyme, Wicklow, Ireland). Two-day-old conidia were harvested, mixed with 2 M KOH for 30 min in an ice water bath, and incubated with 1.2 M sodium acetate buffer (pH 3.8) and GlucazymeTM for 16 h at 40 °C. The solution was resuspended in 10 mL of water, and 10 μL of the solution was incubated with the Megazyme glucose determination reagent (glucose oxidase/peroxidase; GOPOD). The optical density was determined at 510 mm.

For GM production, 5 × 10^7^ conidia were inoculated into 50 mL of modified Brian medium and incubated for 24 h [34]. The extracellular GM content in the supernatants was determined using a GM Ag ELISA kit (MyBioSource, San Diego, CA, USA), following the manufacturer’s instructions.

### 2.8. Preparation of Protein Extracts and Immunoblot Analysis

Phosphorylation of MAPK in AMs induced by conidial challenge was investigated using previous methods [34,40]. Briefly, MH-S cells were starved for 16 h in RPMI 1640 medium without FBS, stimulated with 10-fold conidia, and washed with cold RPMI medium to remove the unbound conidia. After incubation at 37 °C in 5% CO_2_ for different incubation times (3, 5, and 7 h), the cells were washed with cold DPBS and frozen at −70 °C prior to analysis.

Protein extracts were resuspended in RIPA lysis buffer (Elpis Biotech, Lexington, MA, USA) with protease inhibitor cocktail (Calbiochem, San Diego, CA, USA) and phosphatase inhibitor (0.1 M phenylmethylsulfonyl fluoride, 0.5 M sodium fluoride, and 0.1 M sodium orthovanadate), according to the manufacturer’s protocol and previously described method [34]. The lysates were collected using a cell scraper and cleared by centrifugation. Equal amounts of total protein extracts were separated on 10% sodium dodecyl-sulfate polyacrylamide gel electrophoresis (SDS-PAGE) gels and electroblotted onto Hybond-P polyvinylidene difluoride membranes (GE Healthcare, Chicago, IL, USA). The membranes were blocked with 5% skim milk in TBST (20 mM Tris-HCl, pH 7.5, 30 mM NaCl, 0.05% Tween 20) for 2 h and probed with anti-Erk1/2 (1:10,000; Cell Signaling Technology, Danvers, MA, USA), anti-phospho-Erk1/2 (1:10,000; Cell Signaling Technology, Danvers, MA, USA), anti-p38 MAPK (1:10,000; BioLegend, San Diego, CA, USA), anti-phospho-p38 MAPK (1:10,000; Santa Cruz Biotechnology, Dallas, TX, USA), and anti-β-actin (1:10,000; Cell Signaling Technology, Danvers, MA, USA) as primary antibodies and goat anti-rabbit IgG-HRP (1:10,000; Enzo Life Sciences, Farmingdale, NY, USA) and goat anti-mouse IgG-HRP (1:5000; Santa Cruz Biotechnology, Dallas, TX, USA) as secondary antibodies. An ECL chemiluminescence system (Advansta, San Jose, CA, USA) was used for immunological detection. Phosphorylation levels were quantified after normalization to the levels of total ERK and p38 using densitometry scanning and ImageJ (National Institutes of Health, Bethesda, MD, USA).

### 2.9. Cytokine Measurements

For in vitro cytokine expression, 10^6^ MH-S cells were co-cultured with conidia at a conidium-to-macrophage ratio of 10:1 for 6 h, and culture supernatants were frozen at -70 °C, as previously described, with some modifications [34]. The TNF-α concentration in supernatants was measured with ELISA using a mouse TNF-α detection kit (Invitrogen, Waltham, MA, USA) and is presented as the average value (pg/mL) for biological replicates.

### 2.10. Zebrafish Infection Assay by Conidial Microinjection

Zebrafish larvae (*foxn1*/Casper mutants) were obtained from the Zebrafish Center for Disease Modeling (Daejeon, Korea) [33]. All experiments using zebrafish were conducted according to protocols approved by the Animal Ethics Committee of Chungnam National University (202012A-CNU-170). The survival rate of the zebrafish was determined using a previously described method [34]. Briefly, 3 days post-fertilization, larvae were anesthetized and embedded in 1.5% low-melting agarose. Conidia at a concentration of 10^8^ conidia/mL were mixed in a ratio of 1:1 with fluorescein isothiocyanate–dextran (Sigma-Aldrich, St. Louis, MO, USA) for clear visualization of injection success and injected into the common cardinal vein/duct of Cuvier. After injection of the conidial suspension, the infected larvae were incubated in fresh water at 30 °C. For survival analysis, infected larvae were monitored daily and mortality was recorded.

### 2.11. Mouse Strains

CGD (gp91^phox−/−^) mice were obtained from Jackson Laboratory. All animal handling and experimental procedures were performed in accordance with the recommendations of the Guide for the Care and Use of Laboratory Animals of the National Institutes of Health. This study was approved by the Institutional Animal Care and Use Committee of the host campus of Indiana University School of Medicine–Terre Haute, Indiana State University.

### 2.12. Fungal Aspiration and Lung Harvest

Isoflurane-anesthetized mice involuntarily aspirated 10^6^ conidia in 50 μL of suspension. For survival tests, infected mice were monitored for 15 d post-infection (dpi). Mouse bronchoalveolar lavage (BAL) cells and lungs were harvested after 5 d, as previously described [41]. Infected mice were euthanized with sodium pentobartial [42]. The lung samples were perfused with 10 mL of PBS and lyophilized.

### 2.13. Fungal Burden Assay

To quantify the fungal burden, harvested lungs were rapidly frozen in liquid nitrogen. Genomic DNA was extracted from 50 to 100 mg of freeze-dried, homogenized lung tissue using a previously described DNA extraction buffer for *Aspergillus* nucleic acids with subsequent phenol/chloroform extraction [43]. A qPCR fungal burden assay was performed using 1 μg of genomic DNA with 18S rRNA-encoding DNA primers and probe sets with a modified probe quencher (50-/56-FAM/AGC CAG CGG/ZEN/CCC GCA AAT G/3IABkFQ/-30). Samples were amplified in triplicate, with at least four biological replicates from different mice. qPCR was performed using the CFX ConnectTM Real-Time System. Threshold values were used to calculate the corresponding fungal DNA content in the lung tissues.

### 2.14. RNA Extraction from Mice Lungs for Cytokine Analysis

Total RNA was extracted from the lungs and homogenized using TRIzol reagent (Invitrogen, Waltham, MA, USA). Following the aqueous upper phase separation, further RNA purification was performed using a Qiagen RNeasy column with on-column DNase treatment, according to the manufacturer’s recommendations. Mouse β-actin was used as the housekeeping gene for cytokine analysis in the murine model. Most primers were designed using the Primer3 software (version 0.4.0) from the whole sequence available in GenBank.

### 2.15. Flow Cytometric Analysis of BAL Cells

BAL cells were centrifuged, the supernatant was removed, and the cell pellet was resuspended and washed in 1 mL FACS buffer (PBS, 5% FBS, 0.05% sodium azide). The washed pellet was resuspended in a blocking solution containing 10% donkey serum, Fc receptor blocking Ab (clone 24G2), and 1% BSA in PBS and stained with the following antibodies (BD Biosciences, San Jose, CA, USA or eBioscience, San Diego, CA, USA): rat anti-mouse Ly6G-PE-Cy7, rat anti-mouse SiglecF-PE, pan-leukocyte rat anti-mouse CD45-PerCP, and rat anti-mouse CD11c-APC. BAL cells were used to assay the population of immune cells with flow cytometric analysis on a Guava EasyCyte HT system, as previously described [41].

### 2.16. Statistical Analysis

The results are presented as the mean ± standard error of the mean (SEM) obtained from at least three independent experiments. Statistical differences were evaluated using unpaired t tests. Differences between the experimental groups were considered significant at *p* < 0.05.

## 3. Results

### 3.1. SvfA Was Involved in Biofilm Formation

**Figure 1:**
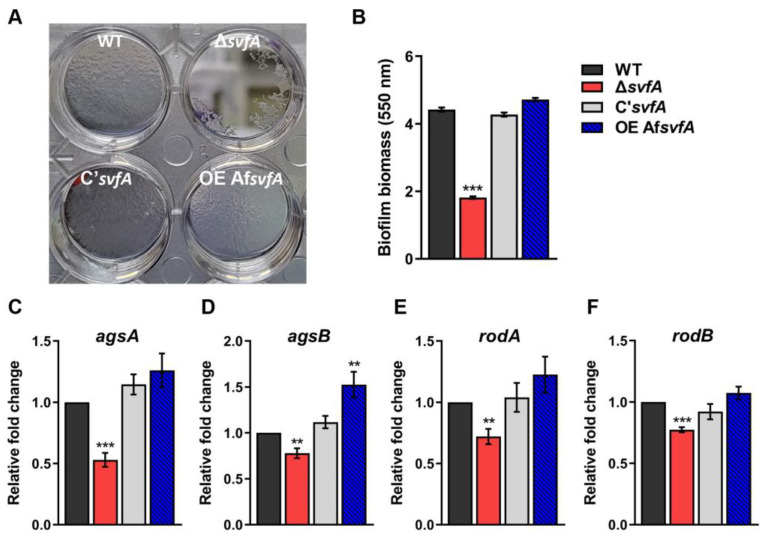
Role of SvfA in *A. nidulans* biofilm formation. Biofilm formation was evaluated after 16 h of growth in GMM. (**A**) Visualization of biofilms attached to the bottom of the individual wells in a 12-well plate. (**B**) Crystal violet staining assay. The biofilms were stained with 0.01% crystal violet and dissolved in 30% acetic acid solution. *N* = 12. (**C**–**F**). Relative expression of α-1,3-glucan synthase genes (*agsA* and *agsB*) and hydrophobin genes (*rodA* and *rodB*). Total RNA was extracted from biofilm cultures. RT-qPCR analysis was performed using 18S rRNA gene as an internal control. *N* = 10. ** *p* ˂ 0.01, *** *p* ˂ 0.001.

One of the characteristics of fungi is the presence of biofilms, which are aggregated in extracellular matrix communities. Biofilm formation alters cytokine production and reduces the killing ability of leukocytes and the recruitment of immune cells [20]. When biofilm formation was evaluated with crystal violet staining, the Δ*svfA* strain showed a 60% reduction compared to the WT and C’*svfA* strains (Figure 1A,B). In both in vivo (aspergilloma and invasive aspergillosis) and in vitro biofilm models, GM, GAG, α-1,3-glucan, melanin, and hydrophobins are the major components of the ECM [19,44]. Under biofilm conditions, α-1,3-glucan synthase genes (*agsA* and *agsB*) and hydrophobin genes (*rodA* and *rodB*) showed significantly reduced expression in Δ*svfA* cultures, whereas the OEAf*svfA* strain showed increased biofilm formation and expression of both genes (Figure 1C–E). These results suggest that SvfA plays a role in biofilm formation by regulating the gene expression of ECM components, including polysaccharides and surface proteins, required for adherence ability in *Aspergillus* species.

### 3.2. SvfA Modulated Protease Activity

**Figure 2:**
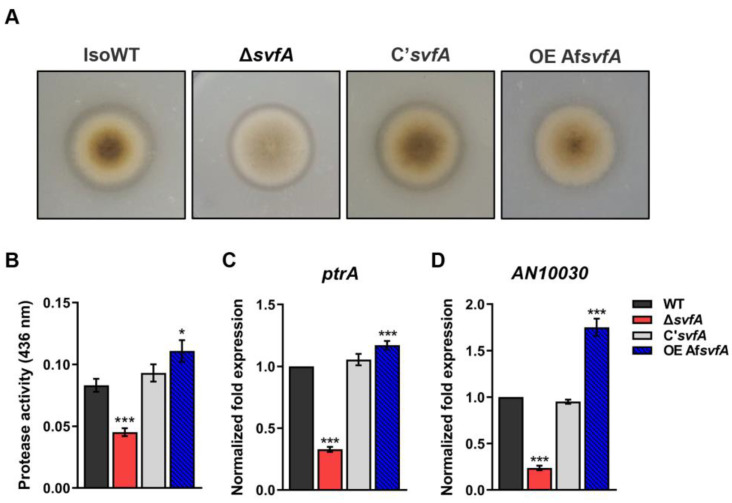
Role of SvfA in protease production. (**A**) Halo formed at edge of colonies of WT, Δ*svfA*, C’*svfA*, and OEAf*svfA A. nidulans* strains. Conidia were point-inoculated on Czapek–Dox medium containing 1% skim milk and incubated at 37 °C. (**B**) Quantification of proteolytic activity. Supernatants were obtained from the 3 d cultures in Czapex–Dox broth containing 1% skim milk. Protein concentrations were calculated using a BCA protein assay kit. Protease activity was evaluated using an azocasein assay. *N* = 10. (**C**,**D**) Relative expression of the putative alkaline protease genes *ptrA* and *AN10030*. Total RNA was extracted from mycelial balls incubated in Czapex–Dox broth. RT-qPCR analysis was performed using the 18S rRNA gene as an internal control. *N* = 15–18. * *p* ˂ 0.05, *** *p* ˂ 0.001.

Secreted proteases are important for nutrient recycling and virulence in many fungi. Proteases are required to evade host recognition and invasion [45]. We examined the possible role of SvfA in controlling the protease activity of *A. nidulans*. When the cultures were grown on 5% skim milk agar, a decrease in the proteolytic activity of the Δ*svfA* strain was observed by the degradation of halos at the edge of the colonies (Figure 2A). Quantitative analysis of azocasein revealed a 46% decrease in protease activity of the Δ*svfA* cultures and a 33% increase in that of the OEAf*svfA* cultures with respect to that of the WT and C’*svfA* strains (Figure 2B). Alkaline protease (Alp1), also known as Asp13 in *A. fumigatus*, provokes airway hyper-responsiveness in asthma [25] and is the most powerful alkaline protease essential for both carbon and nitrogen acquisition [46]. PtrA in *A. nidulans*, an ortholog of *A. fumigatus* Alp1 with a high degree of similarity, is thermostable and shows activity over a broad alkaline pH range [47]. Using protein information from the FungiDB database [48], we identified *AN10030* as an ortholog of *A. fumigatus alp2* gene. The expression levels of both *ptrA* and *AN10030* were reduced in the Δ*svfA* strain and increased in the OEAf*svfA* strain compared to those in the WT and C’*svfA* strains (Figure 2C,D). Taken together, these results indicate that SvfA is involved in protease activity by affecting transcriptional regulation and enzymatic activity.

### 3.3. The Conidia of ΔsvfA were Susceptible to Killing by AMs

**Figure 3:**
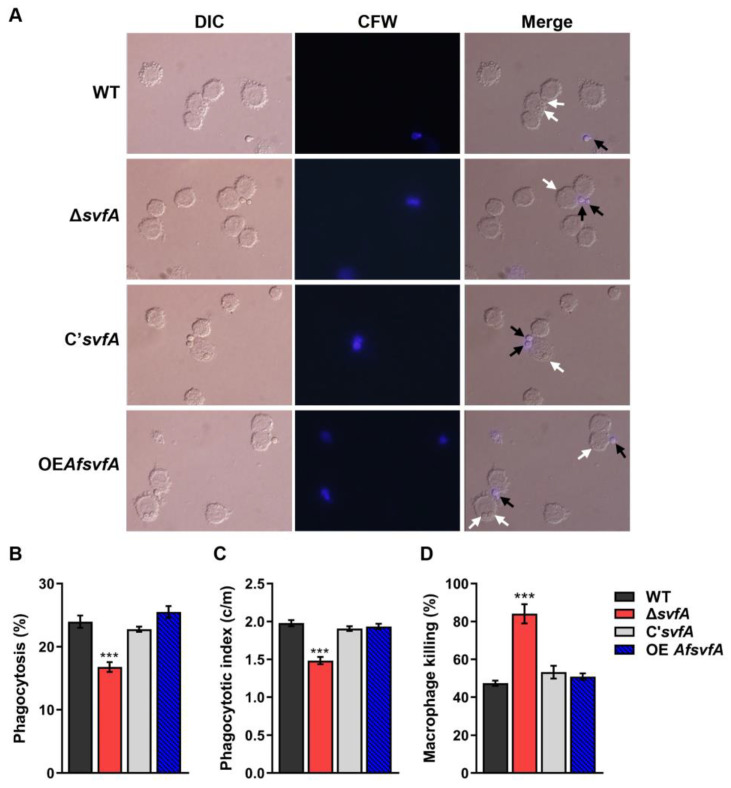
Alveolar macrophage response to Δ*svfA* conidia. MH-S murine alveolar macrophage cells were challenged with a three-fold concentration of *A. nidulans* conidia and incubated for 5 h at 37 °C in an atmosphere of 5% CO_2_. (**A**) Microscopic analysis of the uptake of conidia by the macrophages. External conidia (black arrows) were stained with calcofluor white. White arrows indicate conidia endocytosed by macrophage. (**B**) Phagocytosis of conidia. The percentage of macrophages containing more than one ingested conidia was counted. *N* = 70–150. *** *p* ˂ 0.001. (**C**) Phagocytic index. The average number of ingested conidia per macrophage (c/m). *N* = 230–400. *** *p* ˂ 0.001. (**D**) Macrophage killing assay. MH-S cells were stimulated with 10-fold conidia. The cells were incubated for 0 and 16 h at 37 °C in 5% CO_2_. After incubation, macrophages were lysed, serially diluted and plated on GMM agar. Colony-forming units (CFUs) were counted following incubation for 3 d at 37 °C. The percentage of killed conidia (the difference between the number of CFUs from the lysate at 0 and 16 h per the number of CFU from the lysate at 0 h) was calculated. *N* = 5–6. *** *p* ˂ 0.001.

AMs are the main phagocytic cells in the lungs that protect against *Aspergillus* infection [49]. To investigate phagocytosis and killing of the conidia by AMs, MH-S murine AMs were co-incubated with *A. nidulans* conidia from the WT, Δ*svfA*, C’*svfA*, and OEAf*svfA* strains. The Δ*svfA* conidia (16.8 ± 0.8%) were less susceptible to phagocytosis compared to the WT (24.0 ± 1.0%) and the C’*svfA* strains (22.8 ± 0.4%) (Figure 3A,B). The Δ*svfA* conidia showed a reduced phagocytic index (1.5 vs. 1.9 c/m), which indicates the average number of conidia in a macrophage (Figure 3A,C). Nevertheless, more Δ*svfA* conidia were killed by AMs (47–53% in the WT, C’*svfA*, and OEAf*svfA* strains but 84% in the Δ*svfA*) (Figure 3D). These data suggest that SvfA deletion in *A. nidulans* affects the interaction between fungal PAMPs and PRRs on AMs and resistance to killing after phagocytic engulfment. 

### 3.4. SvfA Affected the Content of Cell Wall PAMPs

**Figure 4:**
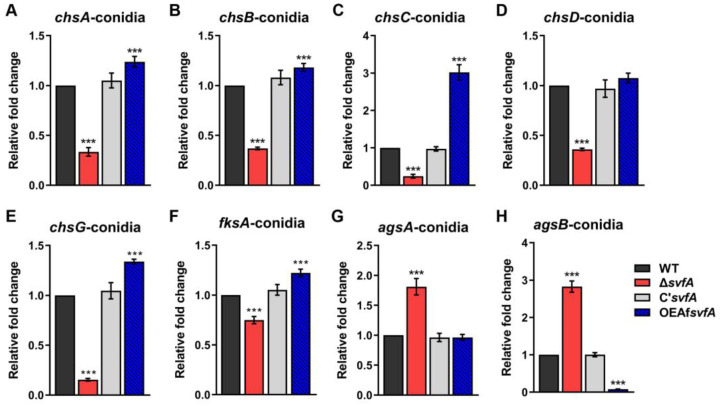
Role of SvfA in the regulation of the expression of cell-wall-associated genes. Conidia were inoculated on GMM, incubated for 2–3 d, and harvested using 0.08% Tween 80 solution. Total RNA was extracted and used for cDNA synthesis. RT-qPCR analysis was performed using the 18S rRNA gene as an internal control. (**A**–**E**) Expression levels of chitin synthase genes, *chsA*, *chsB*, *chsC*, *chsD*, and *chsG*. *N* = 8–10. (**F**) Expression level of β-1,3-glucan synthase gene, *fksA*. *N* = 9–10. (**G**,**H**). Expression levels of α-1,3-glucan synthase genes *agsA* and *agsB*. *N* = 10. *** *p* ˂ 0.001.

**Figure 5:**
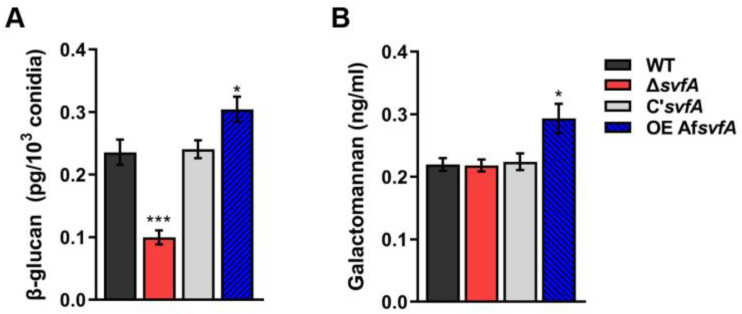
Role of SvfA in biosynthesis of conidia cell wall. (**A**) Amount of β-glucan (pg) per 10^3^ conidia. *N* = 5–9. * *p* ˂ 0.05, *** *p* ˂ 0.001. (**B**) Concentration (ng/mL) of GM. Conidia were inoculated in modified Brian broth and incubated for 24 h at 37 °C. Extracellular GM content in the culture supernatant was assayed with ELISA. *N* = 8–14. * *p* ˂ 0.05.

Many PRRs in host immune cells interact with fungal cell wall components, such as β-1,3-glucan, chitin, proteins, and glycolipids [50]. We investigated the expression of cell wall genes, the products of which are known as fungal PAMPs, such as chitin synthases (*chsA*, *chsB*, *chsC*, *chsD*, and *chsG*), α-1,3-glucan synthase (*agsA*), and β-1,3-glucan synthase (*fksA*), in both vegetative hyphae and conidia [51,52,53]. Decreased expression levels of *chsA*, *chsB*, *chsC*, *chsD*, *chsG*, and *fksA* genes were detected in the Δ*svfA* strain, while the OEAf*svfA* strain showed increased expression levels of these genes compared to the WT strain (Figure 4A–F in conidia and Supplementary Figure S2A–F during vegetative growth). Consistent with the decreased expression of the *fksA* gene, the level of β-1,3-glucan in conidia was significantly reduced in the Δ*svfA* strain and increased in the OEAf*svfA* strain (Figure 5A). These results suggest that SvfA is involved in the expression of chitin and β-1,3-glucan, which are representative PAMPs in the inner cell wall of fungi.

In fungal conidia, α-1,3-glucan, melanin, and hydrophobins form a dense outer layer covering the inner cell wall of PAMPs [23]. The *agsA* and *agsB* genes, encoding α-1,3-glucan synthases, were highly expressed in the Δ*svfA* strain (Figure 4G,H).

Galactomannan (GM) mediates the interaction of *A. fumigatus* conidia with DC-SIGN, which is a C-type lectin receptor mostly expressed in macrophages and dendritic cells [54]. As GM is produced during vegetative growth, we measured GM content in cultures grown in Brian’s medium. While there was no difference among the WT, Δ*svfA*, and C’*svfA* strains, increased GM production was observed in the OEAf*svfA* strain (Figure 5B). This observation suggests that AfSvfA may play a role in GM production. Further experiments are required to reveal that AfSvfA is associated with the production of GM. Collectively, SvfA in *Aspergillus* species affects fungal cell wall components, which could result in the modulation of antifungal immune responses.

### 3.5. Macrophages Challenged with ΔsvfA Conidia Affected ERK Phosphorylation and Produced More TNF-α

**Figure 6:**
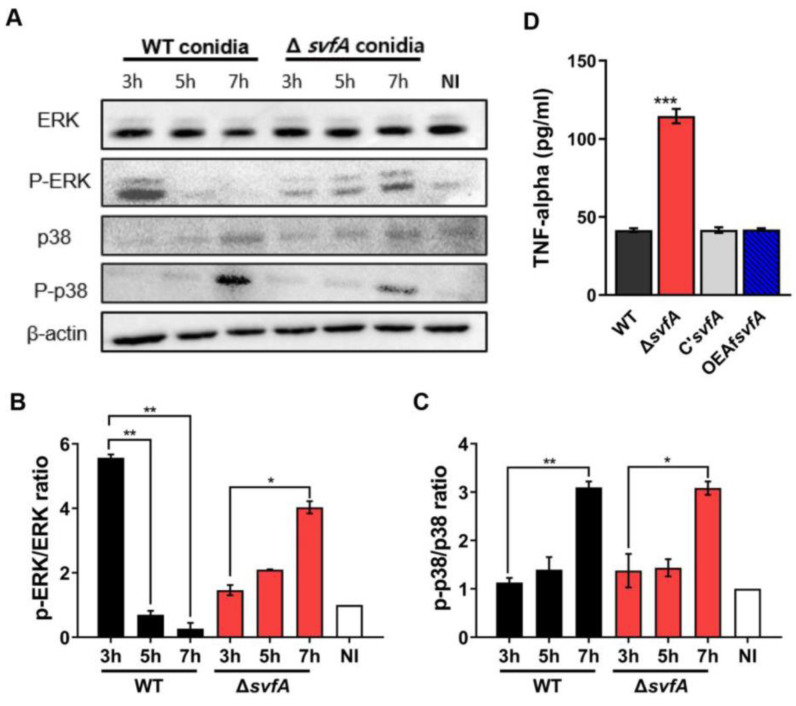
Effect of MAPK phosphorylation and TNF cytokine production from alveolar macrophages challenged with WT and Δ*svfA* conidia. (**A**) Phosphorylation of ERK and p38 after stimulation of MH-S cells with conidia detected with Western blotting. (**B**,**C**). The level of phosphorylation was quantified after normalization to the level of total ERK or p38, respectively. ImageJ software was used. * *p* ˂ 0.05, ** *p* ˂ 0.01. (**D**) Production of TNF-α. Macrophages were challenged with 10-fold conidia and incubated for 6 h. The release of the cytokine was measured with ELISA. *N* = 21–34. *** *p* ˂ 0.001.

Signaling pathways, including ERK and p38, are activated after receptor engagement with PAMPs [55]. We studied the activation of ERK and p38 in vitro using MH-S cells after conidia challenge (Figure 6A). When macrophages were stimulated with WT conidia, ERK was activated at an early time point (5.6 at 3 h) and gradually reduced (0.3 at 7 h) (Figure 6B, WT). After infection with the Δ*svfA* conidia, phosphorylation of ERK gradually increased, and a strong response of ERK was detected at 7 h post-infection (1.5 at 3 h, 2.1 at 5 h, and 4.0 at 7 h) (Figure 6B, Δ*svfA*). However, no significant difference in the phosphorylation patterns of p38 was observed compared to that in cells challenged with WT conidia (Figure 6C).

Activation of this signaling pathway is required for the production of cytokines and chemokines [56]. The pro-inflammatory cytokine TNF-α is produced by conidial infection both in vitro and in vivo [57]. After co-incubation of conidia and MH-S cells for 6 h, cells challenged with the Δ*svfA* conidia showed an increased production of TNF-α (114.6 ± 21.3), compared to the cells with the WT conidia (41.5 ± 1.3) (Figure 6D). These observations are in line with the increased ERK phosphorylation by the Δ*svfA* strain, suggesting that AMs activate the ERK signaling pathway and produce more TNF-α when challenged with Δ*svfA* conidia.

### 3.6. Lack of SvfA Attenuated the Virulence of A. nidulans in the T-Cell-Deficient Zebrafish and the CGD Mice Model

**Figure 7:**
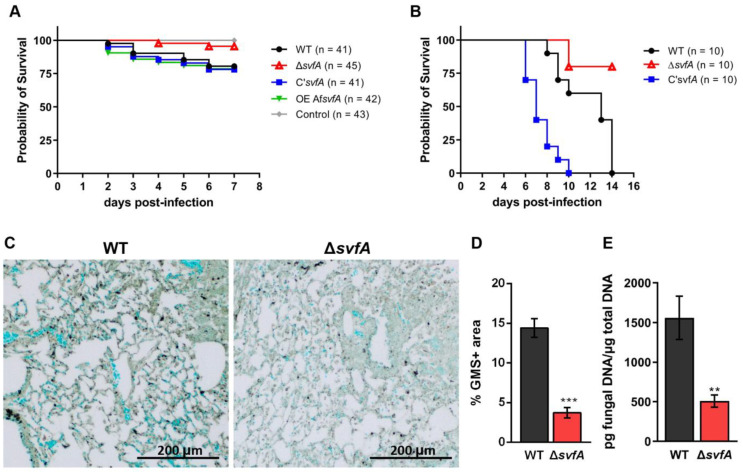
Severity of *A. nidulans* strains in animal models. (**A**) Survival rate of the T-cell-deficient zebrafish larvae (*foxn1* morphant) infected with *A. nidulans* conidia. Conidia were diluted in fluorescent dye to observe the injection site and injected into 3 d post-fertilization larvae (0 dpi). Control represents the injection with only dye. *N* = 41–43/group (**B**) Survival rate of the CGD mice (gp91^phox-/-^ mice) infected with *A. nidulans* conidia. *N* = 10/group. (**C**) Representative lung GMS staining. The lung was harvested from the CGD mice infected with WT or Δ*svfA* conidia at 5 dpi. (**D**) Fungal burden determined from GMS staining of adjacent lung sections. *** *p* ˂ 0.001. (**E**) Fungal burden determined by quantification of fungal DNA from conidia-aspirated lung homogenates. *N* = 4/group. Data are a summary of two independent experiments. ** *p* ˂ 0.01.

Next, we determined the in vivo virulence of the Δ*svfA* strain in two animal models of opportunistic infections. The T-cell-deficient zebrafish model (*foxn1* morphants), a suitable model for studying the innate immune system, has been used for *A. fumigatus* infection [34,58]. Forkhead box protein N1 (Foxn1) encodes an essential transcription factor for thymic epithelial cell development [33]. T-cell-deficient zebrafish were more susceptible to the WT conidia (with approximately 20% mortality) than the Δ*svfA* conidia (with approximately 5% mortality) at 7 dpi (Figure 7A). Furthermore, Δ*svfA* conidia were significantly less virulent in the mouse CGD model. Mice infected with WT conidia and C’*svfA* conidia died in the first 14 and 10 d, respectively, but a clear increase in the survival rate in the Δ*svfA*-conidia-infected mice was observed in 80% of the surviving animals (Figure 7B). These results suggest that SvfA plays a role in the virulence of *A. nidulans*. GMS staining showed decreased hyphal growth from the lungs of the Δ*svfA*-conidia-infected mice (3.7 ± 0.7) compared to the lungs of the WT-conidia-infected mice (14.4 ± 1.2) (Figure 7C,D). In addition, a 67% increase was confirmed using PCR quantification of fungal DNA in the lungs from Δ*svfA*-conidia-infected mice compared to that from WT-conidia-infected mice (Figure 7E).

### 3.7. CGD Mice Infected with the ΔsvfA Conidia Exhibited Decreased Disease Pathology

**Figure 8:**
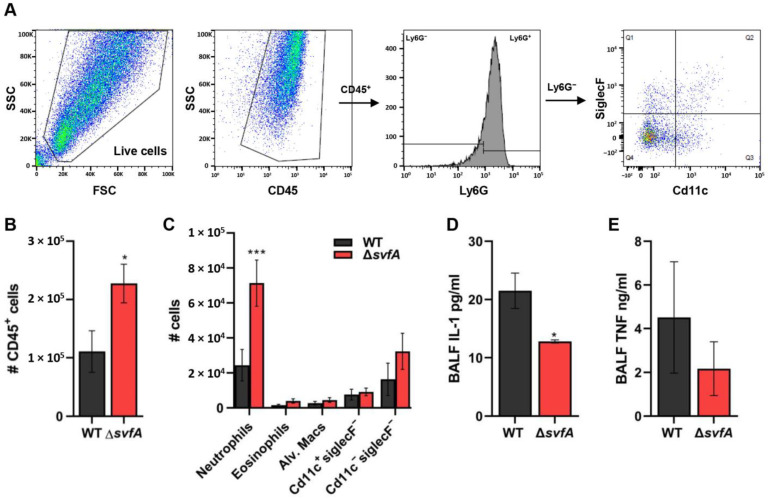
Lung inflammatory responses from the CGD mice after *A. nidulans* aspiration. (**A**). Representative flow cytometric dot plots. BAL cells were isolated from *A. nidulans* WT- and Δ*svfA*-conidia-aspirated mice at 5 d post-infection. The number of cells was determined using flow cytometry. *N* = 6–8/group. (**B**) Total number of CD45^hi^ cells. *N* = 6–8/group. * *p* ˂ 0.05. (**C**) Number of Ly6G^hi^ neutrophils, Ly6G^lo^CD11c^lo^SiglecF^hi^ eosinophils, Ly6G^lo^CD11c^hi^SiglecF^hi^ alveolar macrophages, CD45^hi^Ly6G^lo^CD11c^hi^SiglecF^lo^ cells, and CD45^hi^Ly6G^lo^CD11c^lo^SiglecF^lo^ cells. *N* = 6–8/group. *** *p* ˂ 0.001. (**D**) Interleukin (IL)-1α and (**E**) TNF-α concentration in BAL cells of the CGD mice quantified at the protein level using ELISA. *N* = 4–5/group. Data are a summary of two independent experiments. * *p* ˂ 0.05.

To investigate immune cell recruitment (CD45^hi^Ly6G^hi^ neutrophils, CD45^hi^Ly6G^lo^CD11c^lo^SiglecF^hi^ eosinophils, CD45^hi^Ly6G^lo^CD11c^hi^SiglecF^hi^ alveolar macrophages, CD45^hi^Ly6G^lo^CD11c^hi^SiglecF^lo^ cells, and CD45^hi^Ly6G^lo^CD11c^lo^SiglecF^lo^ cells), BAL cells were harvested at 5 dpi, stained, and analyzed by flow cytometry (Figure 8A). The number of CD45^+^ cells, particularly Ly6G^+^ neutrophils, was increased in the CGD mice infected with the Δ*svfA* conidia compared to that in the mice infected with WT conidia (Figure 8B,C). When we investigated the mRNA expression from the lung homogenates, the expression levels of the IL-12β, IL-17, and IL-22 genes were increased in the mice infected with the Δ*svfA* conidia compared to those in the mice infected with the WT conidia (Supplementary Figure S3). However, the protein level of the pro-inflammatory cytokine IL-1α was decreased in the BAL supernatant from mice infected with the Δ*svfA* conidia (Figure 8D). There was no significant difference in IL-1α, IL-6, and TNF-α mRNA expression in the lung and protein production of TNF-α in BAL (Supplementary Figure S3 and Figure 8E). These results suggest that the deletion of *svfA* in *A. nidulans* results in a modulated immunogenic response during infection.

## 4. Discussion

To investigate the involvement of SvfA in fungal pathogenicity, we tested the role of SvfA in the production of virulence factors, including cell wall PAMPs, biofilms, and proteases, and performed an in vitro phagocytosis assay as well as in vivo survival tests with animal models using the *A. nidulans svfA*-deletion strain.

In this study, we showed that the Δ*svfA* conidia were less recognized by AMs than the WT conidia (Figure 3A,B). Cell walls, which are essential structures for cell viability, morphology, and stress response, mediate interactions with external stimuli, triggering intracellular signaling pathways [59]. Defects in cell wall components influencing fungal virulence have been reported for many pathogenic fungi, including *Candida albicans*, *Cryptococcus neoformans*, and *A. fumigatus* [59,60,61,62]. To investigate if defects in cell wall components by an inhibitor can affect virulence, WT conidia were grown on GMM with Congo red (CR), which binds to β-1,3-glucans and is used for phagocytosis assays [63]. The conidia grown on GMM with CR were less susceptible to phagocytosis and showed a reduced phagocytic index compared to the conidia grown on GMM (Supplementary Figure S4). Also, murine models of experimental invasive pulmonary aspergillosis have documented concentration-dependent activity in neutropenic mice administered caspofungin, an antifungal drug targeting β-1,3-glucan synthesis [64]. Some cell wall PAMPs stimulate cellular and humoral responses during infection [13]. Recognition of β-1,3-glucan, a PAMP, by Dectin-1 is crucial for the activation of LC3-associated phagocytosis in monocytes and macrophages [23]. Our study revealed that SvfA is involved in cell wall synthesis by modulating the transcription of cell wall genes (*chsA*, *chsB*, *chsC*, *chsD*, *chsG*, and *fksA*) (Figure 4A–F). Therefore, changes in cell wall composition, such as the reduction in β-1,3-glucan content in the conidia of the Δ*svfA* strain (Figure 5A), may result in reduced recognition of conidia by AMs by affecting the PAMP–PRR interaction. Notably, in the devastating necrotrophic fungal pathogen *S. sclerotiorum*, *SsSvf1*-gene-silenced strains are sensitive to cell wall damage and show defects in virulence [32].

Unlike most genes for chitin synthases and β-1,3-glucan synthase, the *agsA* and *agsB* genes for α-1,3-glucan synthases were highly expressed in Δ*svfA* conidia (Figure 4G–H). In many fungi, including *A. fumigatus*, the pathogenic dimorphic yeast *Histoplasma capsulatum*, and the rice blast fungus *Magnaporthe grisea*, α-1,3-glucan masks fungal cell wall PAMPs, such as β-1,3-glucan and chitin [65,66,67]. Our data suggest that the increased *agsA* expression in the Δ*svfA* strain supports a stealth role that prevents recognition by the host. Taken together, decreased expression of inner cell wall genes and increased expression of the *agsA* gene, one of the outer cell wall genes, can account for the decreased recognition of Δ*svfA* conidia by AMs.

Under aerial conditions and in patients with *Aspergillus* infection, α-1,3-glucan is responsible for the aggregation of conidia and hyphae for biofilm formation [68]. *A. fumigatus* has three genes (*ags1*, *ags2*, and *ags3*) for α-1,3-glucan synthesis [69,70]; *A. nidulans* has two genes (*agsA* and *agsB*). The *ags1* and *ags2* in *A. fumigatus* are counterparts of *agsB* and *agsA* in *A. nidulans* [71]. AgsB plays a major role in α-1,3-glucan biosynthesis, whereas AgsA plays a minor role [71]. Interestingly, *agsA* and *agsB* expression in the Δ*svfA* strain decreased during biofilm formation (Figure 1C,D), which was also significantly reduced in the Δ*svfA* strain (Figure 1A). In addition, the expression of *rodA* and *rodB* genes was also reduced in the Δ*svfA* strain under biofilm-forming conditions (Figure 1E,F). Hydrophobins are insoluble complexes for rodlets on the surface of the conidia. Both *rodA* and *rodB* for hydrophobin have been reported to be highly expressed in *A. fumigatus* biofilms [72]. These data suggest that SvfA affects the expression of the genes (*agsA*, *agsB*, *rodA*, and *rodB*) that are highly expressed in biofilms, which is attributed to the reduction in biofilm formation.

*Aspergillus* species produce a wide range of proteases and degrading enzymes to digest macromolecules for metabolism, which probably enables nutrition to be obtained during infection [23]. In this study, we investigated the alkaline protease activity. The Δ*svfA* strain showed a decrease in alkaline protease activity with decreased expression of *ptrA* and *AN10030* genes for the putative serine proteases (Figure 2). Based on the fact that the Δ*svfA* strain showed reduced biofilm formation and protease activity, we predicted a decrease in pathogenicity in vivo.

It has been reported that p38 plays a minor role in defense against *A. fumigatus*; activation of p38 was only observed when a high number of *A. fumigatus* conidia were infected, whereas a strong response of ERK was observed with few conidia [17]. When the phosphorylation of ERK and p38 in MH-S cells infected with *A. nidulans* conidia was observed, no significant difference in the phosphorylation of p38 between the macrophages with WT and the Δ*svfA* strain was detected, but the phosphorylation of ERK was gradually increased in macrophages challenged with the Δ*svfA* conidia (Figure 6A–C). NF-κB plays a role in the production of cytokines, such as TNF-α, IL-1, and IL-6 [73]. Translocation of NF-κB is not induced by resting conidia at an early time point but is observed after 6 h of co-incubation with conidia and macrophages [17]. In our study, TNF-α concentration from macrophages 6 h after co-incubation with conidia was significantly increased in macrophages with Δ*svfA* conidia (Figure 6D), and this increase was concomitant with increased ERK phosphorylation in Δ*svfA*-challenged macrophages. The signaling pathways of AMs for the clearance of *Aspergillus* are poorly understood. In vivo experiments suggest that ERK is an essential MAPK in the defense against conidia, whereas the activation of NF-κB appears to play a secondary role [41]. The Δ*svfA* strain altered fungal cell wall components and other virulence factors, such as proteases and morphology. The combined contribution of virulence factors can affect signaling pathways.

After recognition by host receptors, swollen conidia are phagocytosed, but they are able to escape from phagocytes via a two-step mechanism: inhibition of phagolysosomal acidification and anti-apoptotic effects on an intracellular niche [74]. The Δ*svfA* conidia were not recognized by the immune cells as much as the WT conidia, resulting from the change of the cell wall PAMPs. However, once they were recognized and phagocytosed, phagocytes cleared Δ*svfA* conidia easily. Indeed, our previous study revealed that the *svfA*-deletion strain shows retarded growth and increased sensitivity to oxidative stress [26], which can make the Δ*svfA* strain susceptible to killing by macrophages. In addition, the Δ*svfA* strain showed reductions in many other virulence factors except phagocytosis and phagocytic index. The Δ*svfA* strain also elicited a host immune response more strongly that the WT strain. These results suggest that Δ*svfA* conidia are susceptible to phagolysosomal acidification, which could be explained by structural or qualitative changes in the conidial cell wall and decreased resistance to oxidative stress. SvfA is required for the survival of yeast and fungi under oxidative stress [26,28,32]. In *S. cerevisiae*, Svf1 is required for survival under oxidative and cold-stress conditions [28]. In the plant fungal pathogen *S. sclerotiorum*, *SsSvf*-gene-silenced strains showed inhibited hyphal growth under oxidative conditions (H_2_O_2_ and menadione), impaired cell wall integrity, and reduced virulence [32]. In another fungal pathogen, *F. graminearum*, an *FgSvf1-*deletion mutant shows reduced resistance to osmotic, fungicide, and cold stress and reduced oxidative stress sensitivity and pathogenicity [31].

Our results suggest that both increased conidial killing by macrophages and reduced biofilm formation and protease activity in the Δ*svfA* strain resulted in attenuated virulence in an in vivo model system. The T-cell-deficient zebrafish model, which lacks an adaptive immune system, is suitable for studying innate immune responses [58]. The virulence of the Δ*svfA* strain (95% survival at 7 dpi) was decreased in a T-cell-deficient zebrafish infection model compared to that of the WT strain (80% survival at 7 dpi) (Figure 7A). In our previous study, *A. fumigatus* WT-conidia-infected zebrafish showed approximately 50% survival at 8 dpi in T-cell-deficient zebrafish [34]. We hypothesized that in the CGD model, the virulence of *A. nidulans* could be clearly investigated. While the CGD mice with the Δ*svfA* strain showed 80% survival, all the mice with WT or C’*svfA* were dead at 14 and 10 dpi, respectively (0% survival) (Figure 7B). These results are in agreement with previous studies on the virulence of *A. nidulans* using the CGD model, which showed a significantly decreased survival rate [75].

In the lungs of CGD mice infected with the Δ*svfA* strain, a reduced fungal burden was observed with an increased number of neutrophils and reduced IL-1α production (Figure 7C–E and Figure 8). The ability to produce IL-1α and TNF-α is enhanced under hypoxia caused by tissue damage [76]. Pulmonary hypoxia is a commonly observed phenomenon in in vivo aspergillosis models [77]. Neutrophils and AMs are essential for clearing *Aspergillus* conidia from the lungs [78]. In mouse models of CGD, increased production of pro-inflammatory cytokines was observed in the lungs following intratracheal inoculation with *A. fumigatus* [79]. The Δ*svfA* conidia infection did not result in a difference in the number of AMs but showed aberrant recruitment of neutrophils (Figure 8C). In in vivo systems, neutrophils are recruited to the site of infection by responding to various chemokines, peptides, and chemicals released by pathogens. Although CGD mice displayed impaired recruitment and function of neutrophils [80], the changes caused by SvfA deletion resulted in excessive recruitment of neutrophils. However, in line with the data showing decreased fungal growth in the lungs of the ∆*svfA*-conidia-infected mice in Figure 7, IL-1α and TNF-α pro-inflammatory cytokine production was reduced in the BAL cells of the mice infected with the Δ*svfA* conidia (Figure 8D,E). In contrast, fungal burden from the lungs in WT-conidia-infected mice was high, suggesting that cytokine production increased in response to the fungal pathogen to clear them. This indicated that the Δ*svfA* strain caused different immunogenic responses, which could be associated with alterations in cell wall structure or protease activity.

Thus, SvfA is required for the biosynthesis of cell wall PAMPs, biofilm formation, and protease activity, which affect fungal pathogenicity. This study reveals the roles of *A. nidulans* SvfA in virulence and suggests the possibility of the similar role of *A. fumigatus* AfSvfA.

## Data Availability

Not applicable.

## Supplementary Materials

The following supporting information can be downloaded at: https://www.mdpi.com/article/10.3390/jof9020143/s1, Figure S1: Functional complementation of Δ*svfA* by overexpression of Af*svfA* gene; Figure S2: Expression level of chitin synthases (*chsA*, *chsB*, *chsC*, *chsD*, and *chsG*), β-1,3-glucan synthase (*fksA*), and α-glucan synthase (*agsA* and *agsB*) during vegetative growth; Figure S3: Expression level of cytokine genes. mRNA was harvested from mouse lung homogenates and analyzed with RT-qPCR for expression of the indicated cytokines. Figure S4: Alveolar macrophage response to WT conidia grown on GMM containing 200 μg/mL of Congo red (CR). Table S1: Primers used in this study.
