# Supplementary material for: Survival Factor A (SvfA) Contributes to Aspergillus nidulans Pathogenicity
Source: J Fungi (Basel). 2023 Jan 21;9(2):143. doi: 10.3390/jof9020143 (PMC9962611; doi:10.3390/jof9020143)
Supplement: Supplementary file 1 [file jof-09-00143-s001.zip › JoF-ANsvfA-Supple 0101.pdf]

Supplementary information

Survival factor A (SvfA) contributes to Aspergillus nidulans pathogenicity

Joo-Yeon Lim[1], Ye-Eun Jung[2], Hye-Eun Hwang[3], Cheol-Hee Kim[3], Nese Basaran Akgul[1], Sri Harshini Goli[1], Steven P. Templeton[1]* and Hee-Moon Park[2]*

[1] Department of Microbiology and Immunology, Indiana University School of Medicine-Terre Haute, Terre Haute, IN; jooyeonlim1220@gmail.com (JY Lim), nbasaran@iu.edu (NB Akgul), sgoli@sycamores.indstate.edu (SH Goli) and and sptemple@iupui.edu (S Templeton)

[2] Laboratory of Cellular Differentiation, Department of Microbiology and Molecular Biology, College of Bioscience and Biotechnology, Chungnam National University, Daejeon 34134, Republic of Korea; jye6865@naver.com (YE Jung)

[3] Laboratory of Developmental Genetics Department of Biology, College of Bioscience and Biotechnology, Chungnam National University, Daejeon 34134, Republic of Korea; gwj03068@naver.com (HE Hwang) and zebrakim@cnu.ac.kr (CH Kim)

* To whom correspondence should be addressed.

Hee-Moon Park, Tel: 042-821-7553/ Fax : 042-822-7367, E-mail: hmpark@cnu.ac.kr

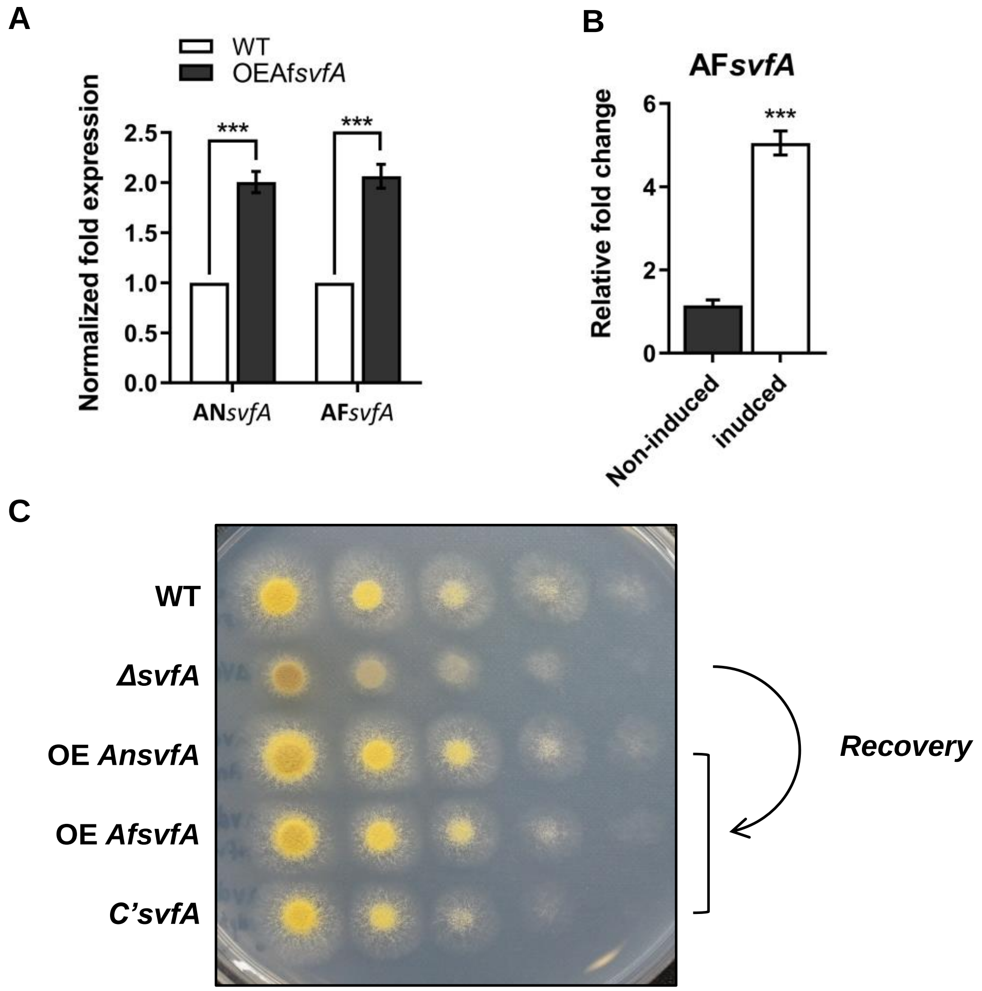

Supplementary Figure S1. Functional complementation of Δ*svfA* by overexpression of *AfsvfA* gene. A. Expression level of *ANsvfA* and *AFsvfA* genes in WT and OE*AfsvfA* strains. Spores were inoculated in GMM liquid broth with 0.6% sodium nitrate. ***P < 0.001. B. Expression level of *AFsvfA* gene in OE*AfsvfA* strain grown in non-inducing media (GMM containing 0.2% ammonium tartrate as a nitrogen source) or inducing media (GMM containing 0.6% sodium nitrate as a nitrogen source). Total RNA was extracted and RT-qPCR analysis was performed using 18S rRNA gene as an internal control. ***P < 0.001. C. Serial diluted conidia from WT, Δ*svfA*, OE*svfA*, OE *AfsvfA* and C'*svfA* were inoculated on GMM including 0.6% sodium nitrate and incubated for 2 days at 37°C.

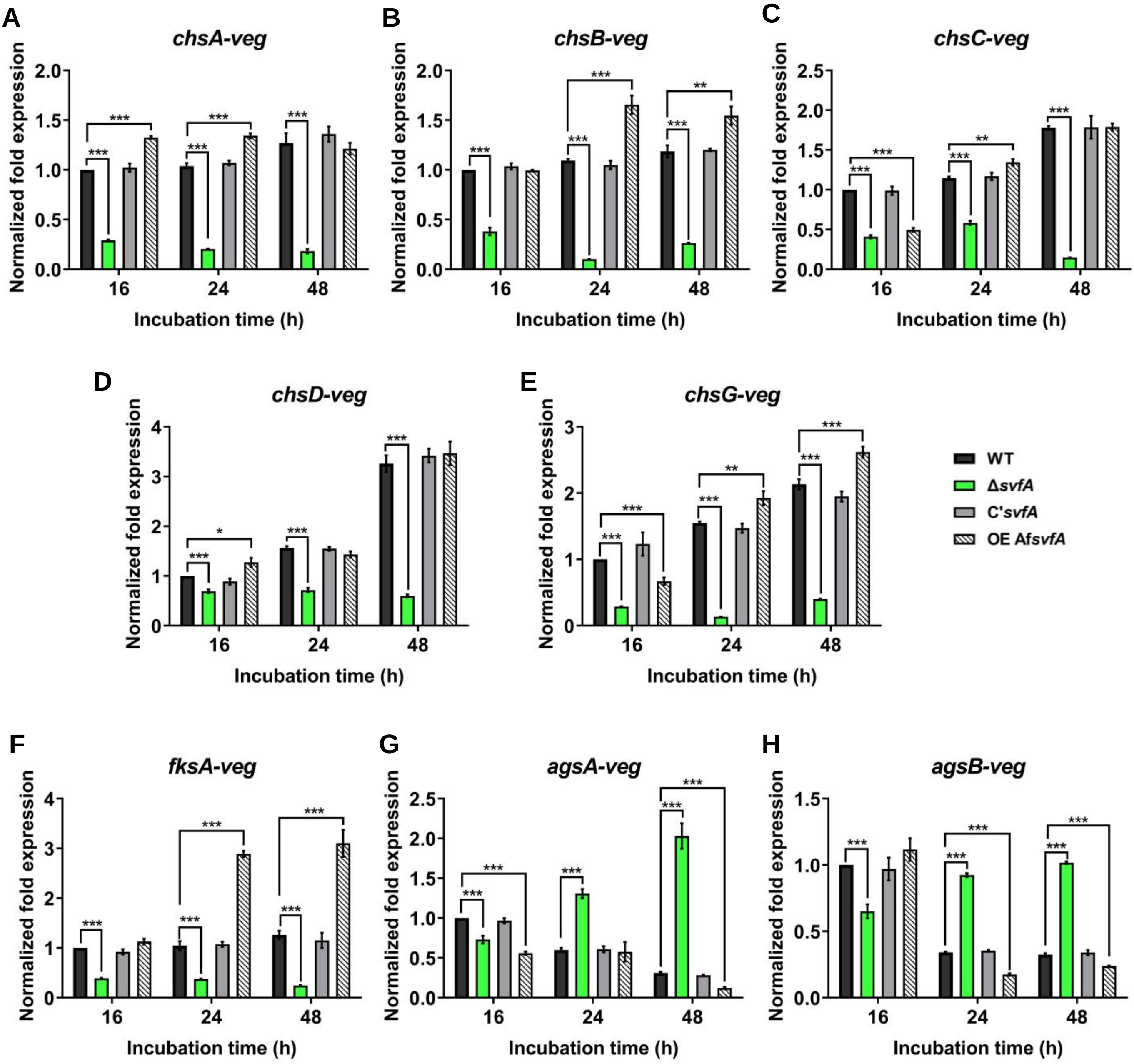

Supplementary Figure S2. Expression level of chitin synthases (*chsA, chsB, chsC, chsD,* and *chsG*), β-1,3-glucan synthase (*fksA*) and α-glucan synthase (*agsA* and *agsB*) during vegetative growth. Spores were inoculated in MM liquid broth and incubated for the indicated time. Total RNA was extracted and RT-qPCR analysis was performed using 18S rRNA gene as an internal control. **P < 0.01, ***P < 0.001

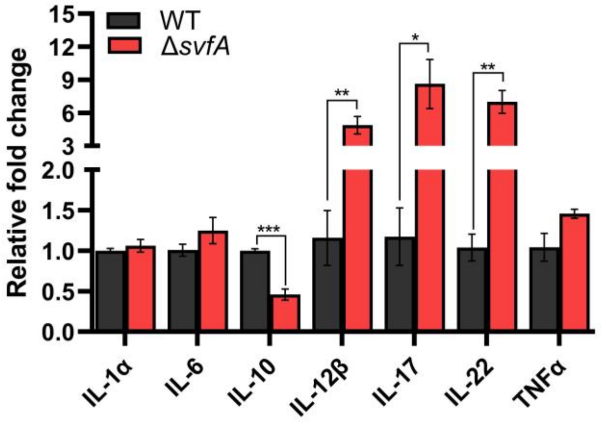

Supplementary Figure S3. Expression level of cytokine genes. mRNA was harvested from mouse lung homogenates and analyzed by RT-qPCR for expression of the indicated cytokines. Expression is indicated as a fold change above WT expression. *$P < 0.05$, **$P < 0.01$, ***$P < 0.001$.

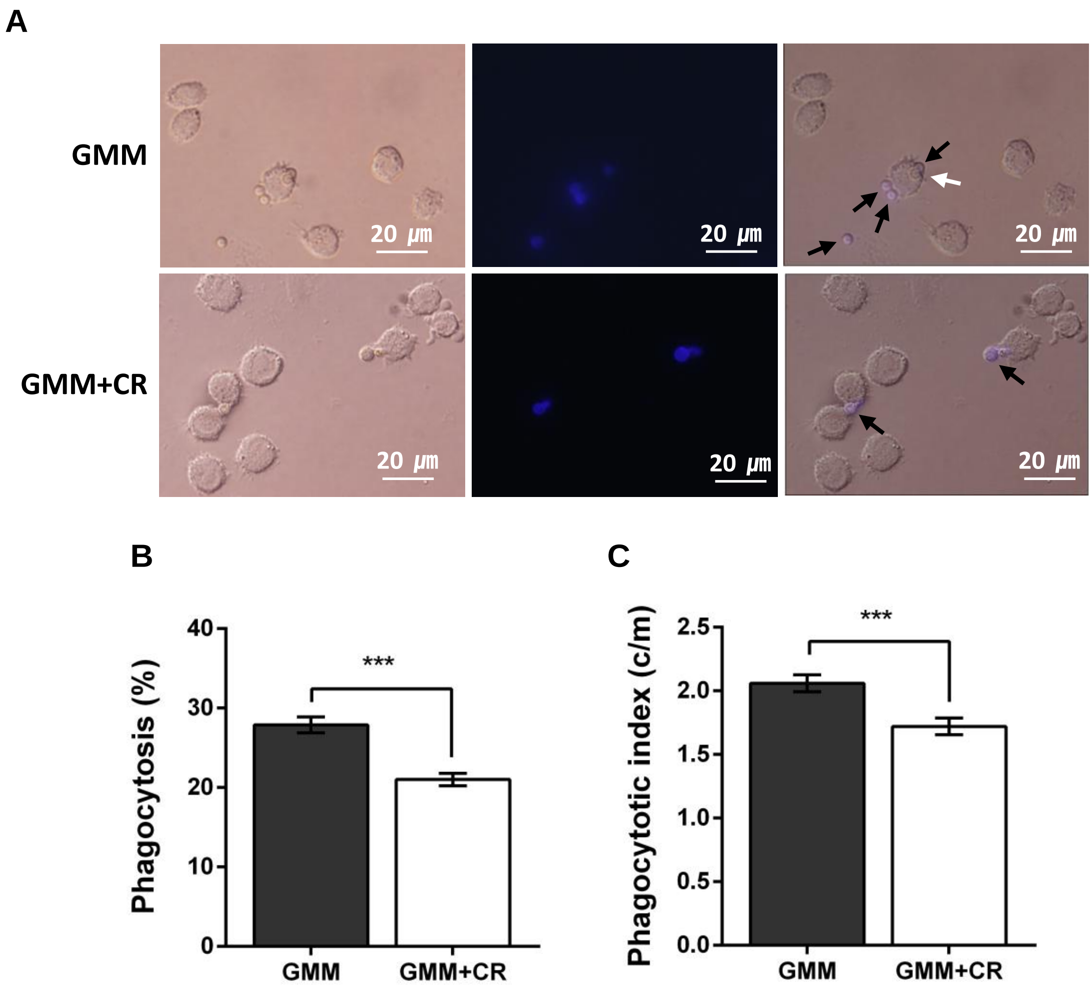

Supplementary Figure S4. Alveolar macrophage response to WT conidia grown on GMM containing 200 μg/mL of Congo red (CR). MH-S murine alveolar macrophage cells were challenged with a three-fold concentration of *A. nidulans* conidia and incubated for 5 h at 37˚C in an atmosphere of 5% CO_2. (A) Microscopic analysis of the uptake of conidia by the macrophages. External conidia (black arrows) were stained by calcofluor white. White arrows indicate conidia endocytosed by macrophage. (B) Phagocytosis of conidia. The percentage of macrophages containing more than one ingested conidia was counted. ***P < 0.001. N = 67–100. (C) Phagocytic index. The average number of the ingested conidia per macrophage (c/m). N = 154–185. ***P < 0.001.
